# PTFOS: Flexible and Absorbable Intracranial Electrodes for Magnetic Resonance Imaging

**DOI:** 10.1371/journal.pone.0041187

**Published:** 2012-09-12

**Authors:** Giorgio Bonmassar, Kyoko Fujimoto, Alexandra J. Golby

**Affiliations:** 1 A. A. Martinos Center, Harvard Medical School, Massachusetts General Hospital, Charlestown, Massachusetts, United States of America; 2 Departments of Neurosurgery and Radiology, Brigham and Women's Hospital, Harvard Medical School, Boston, Massachusetts, United States of America; University of Minnesota, United States of America

## Abstract

Intracranial electrocortical recording and stimulation can provide unique knowledge about functional brain anatomy in patients undergoing brain surgery. This approach is commonly used in the treatment of medically refractory epilepsy. However, it can be very difficult to integrate the results of cortical recordings with other brain mapping modalities, particularly functional magnetic resonance imaging (fMRI). The ability to integrate imaging and electrophysiological information with simultaneous subdural electrocortical recording/stimulation and fMRI could offer significant insight for cognitive and systems neuroscience as well as for clinical neurology, particularly for patients with epilepsy or functional disorders. However, standard subdural electrodes cause significant artifact in MRI images, and concerns about risks such as cortical heating have generally precluded obtaining MRI in patients with implanted electrodes. We propose an electrode set based on polymer thick film organic substrate (PTFOS), an organic absorbable, flexible and stretchable electrode grid for intracranial use. These new types of MRI transparent intracranial electrodes are based on nano-particle ink technology that builds on our earlier development of an EEG/fMRI electrode set for scalp recording. The development of MRI-compatible recording/stimulation electrodes with a very thin profile could allow functional mapping at the individual subject level of the underlying feedback and feed forward networks. The thin flexible substrate would allow the electrodes to optimally contact the convoluted brain surface. Performance properties of the PTFOS were assessed by MRI measurements, finite difference time domain (FDTD) simulations, micro-volt recording, and injecting currents using standard electrocortical stimulation in phantoms. In contrast to the large artifacts exhibited with standard electrode sets, the PTFOS exhibited no artifact due to the reduced amount of metal and conductivity of the electrode/trace ink and had similar electrical properties to a standard subdural electrode set. The enhanced image quality could enable routine MRI exams of patients with intracranial electrode implantation and could also lead to chronic implantation solutions.

## Introduction

Knowledge about functional brain anatomy in patients undergoing brain surgery may be acquired through subdural electrocortical recordings and stimulation [1]. This approach is commonly used in the treatment of medically refractory epilepsy [2], [3]. However, it can be very difficult to integrate the results of subdural recordings with other brain mapping modalities, particularly functional magnetic resonance imaging (fMRI) [4]. The ability to integrate imaging and electrophysiological information with simultaneous subdural electrocortical recording/stimulation and fMRI could offer significant insight for cognitive and systems neuroscience as well as for clinical neurology and neurosurgery, particularly for patients with epilepsy or movement disorders. However, standard subdural electrodes cause significant artifact in MRI images, and concern about risks such as cortical heating have generally precluded obtaining MRI in patients with implanted electrodes.

**Figure 1:**
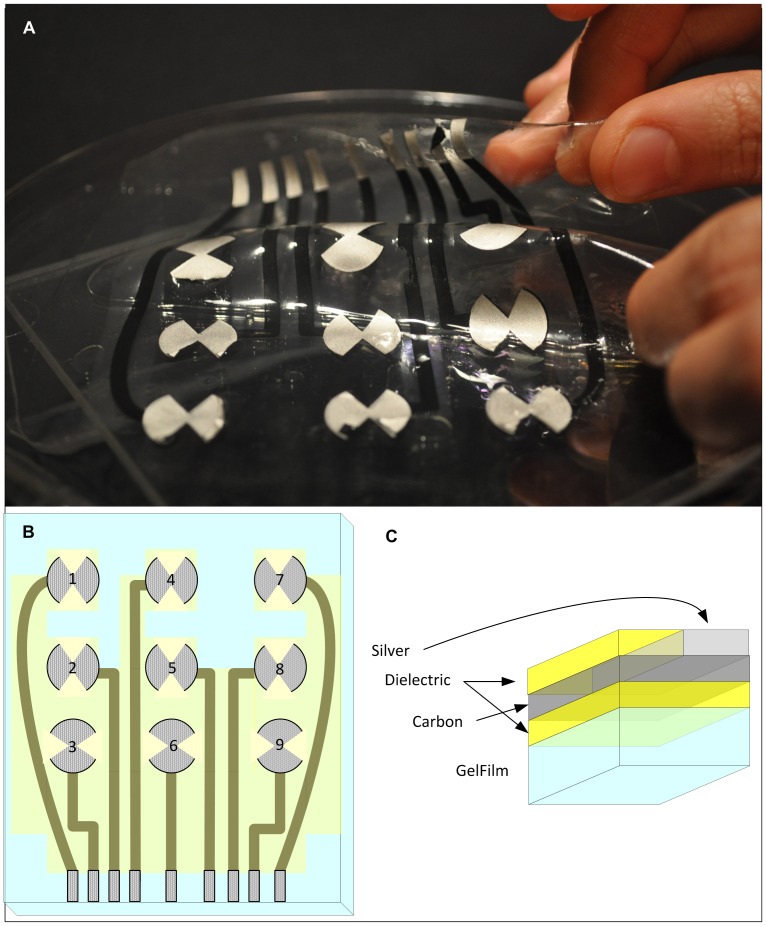
The polymer thick film organic substrate (PTFOS) flexible electrode set. (**A**) Image, (**B**) layout and (**C**) sectional cut of the PTFOS. The 4 layers are made of dielectric, carbon, silver and Gelfilm.

In this paper we describe an MRI-compatible organic electrode set of absorbable, flexible and stretchable material for intracranial use (**Fig. 1A**) based on our earlier development of an EEG/fMRI electrode set for scalp recording [5]. There is a growing interest in flexible and stretchable electronics [6] made possible by the recent development of organic-based conductors and metallic nanostructures. A thin flexible substrate would allow the electrodes to optimally contact the convoluted brain surface and would create less distortion of the brain while they are in place, potentially allowing longer term use with implications for the development of brain-machine interfaces [7], [8]. Furthermore, the development of MRI-compatible electrodes with a very thin profile could allow functional mapping and modulation at the individual subject level of the underlying feedback and feed forward networks [6], [9].

**Figure 2:**
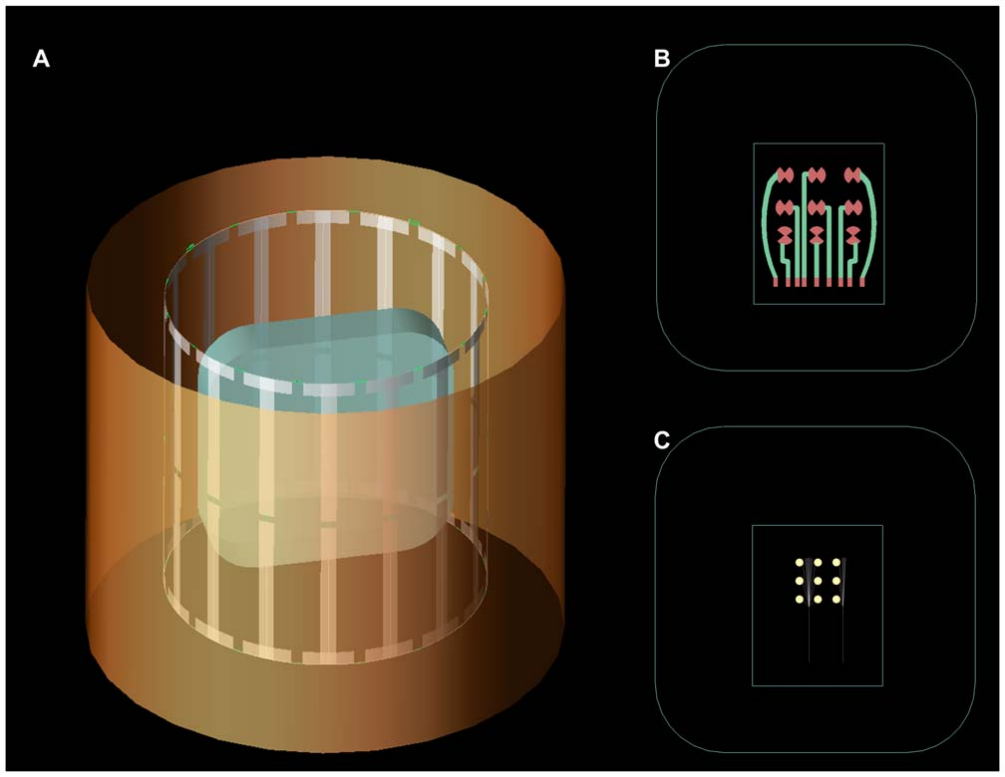
The geometric model used in the electromagnetic simulations. (**A**) The shield, MRI birdcage coil and phantom model used in the Finite Differences Time Domain (FDTD) simulations. Detail model of the (B) PTFOS electrode set and (C) the standard Platinum electrode set.

A challenge in creating electrodes for use in MRI is that the presence of metal can cause distortion due to: (1) susceptibility differences between the subdural grid and the tissue and (2) modification of the radiofrequency (RF) fields in the head induced by RF currents in the metal. The susceptibility difference between the metal the surrounding brain parenchymal tissue causes a local magnetic field inhomogeneity which is proportional to the static field strength (or B_0_), resulting in both a local change in precession (Larmor) frequency and in a shorter relaxation time T_2_ [10]. The reduction in T_2_ causes local dephasing, which affects more pronouncedly the fMRI sequences. In general, the susceptibility distortion in the tissue leads to spatial mismapping of information and may produce MRI signal loss near the implant. We aimed to reduce susceptibility artifacts by using conductive polymers that contain only a minimum amount of metallic particles. The second type of MRI artifact is a variation in intensity across the image due to a lack of a perfectly uniform magnetic component of the RF (or **B_1_**) field caused by the presence of RF induced currents in the highly conductive wires and electrodes of the subdural grids [11]. Electromagnetic numerical (or finite difference time domain (FDTD)) simulations (**Fig. 2**) allowed us to quantify the **B_1_** field changes generated by this type of distortion, which have been reduced by introducing the proper conductive polymers in the proposed electrode set. Overall performance properties of the PTFOS electrodes were assessed by MRI phantom measurements and FDTD simulations.

The electrodes were constructed (**Fig. 1B and 1C**) by Polymer Thick Film (PTF) deposition on top of an organic substrate (PTFOS) absorbable gelatin film (Gelfilm by Pharmacia and Upjohn Co, Division of Pfizer Inc., NY), which is commonly used in neurosurgery as a dural substitute. The Gelfilm is manufactured from denaturized collagen and is absorbed within a few months thus leaving electrodes/traces with a width of only a few microns, which is the typical width of printed traces. Polymer thick film (PTF) technology is widely used in the electronic manufacturing industry in the production of electronic solid state components such as PTF-based blood oxygen sensors, pH sensors, and disposable electrodes for electrophysiology. In the medical industry, PTF is often the technology of choice because of the availability of non-hazardous polymers and the ease of producing disposable devices.

## Methods

### Electrode construction

Two separate, but geometrically comparable intracranial electrode sets were studied; the first was the PTFOS set (**Fig. 1A and 1B**) that consisted of electrodes made of silver ink and leads made of carbon ink isolated by a dielectric and the second were platinum and a stainless commercial subdural electrode sets (Ad-Tech Medical Instruments Corp., Racine WI) we will refer to as the standard set. The PTFOS electrodes (**Fig. 1C**) were fabricated by laying three different PTF layers on 100 mm×125 mm Gelfilm. The following PTF materials (Creative Materials Inc., Tyngsboro, MA) were deposited: 118–43 (silver), 119–28 (carbon) and 118–02 (dielectric), with traces resistance after construction as specified in **Table 1**. The PTFOS electrode design (**Fig. 1B**) consisted of cut disks to reduce eddy currents, since the missing sectors break the circular symmetry of electrodes and attenuate the eddy currents. The approximate PTFOS electrode area was 265 mm2 compared to the 78 mm2 of the standard electrodes allowed for a worst case comparison between PTFOS and commercial with respect to MRI artifacts. Furthermore, a larger area of the PTFOS allowed for observing similar currents and voltages when the electrodes were used in the voltage or current source experiments for comparison with the commercial set.

**Table 1:** Resistivity @100Hz of each electrode/lead.

Electrode #	1	2	3	4	5	6	7	8	9
R (kΩ)	3.1	1.4	1.9	2.7	1.4	2.8	2.4	1.8	4.0 (*)

Numbers refer to drawings in **Fig. 1**. (*) Electrode 9 was retested after 3 months in saline solution and exhibited a 5.1 kΩ resistivity.

### MRI Measurements

**Figure 3:**
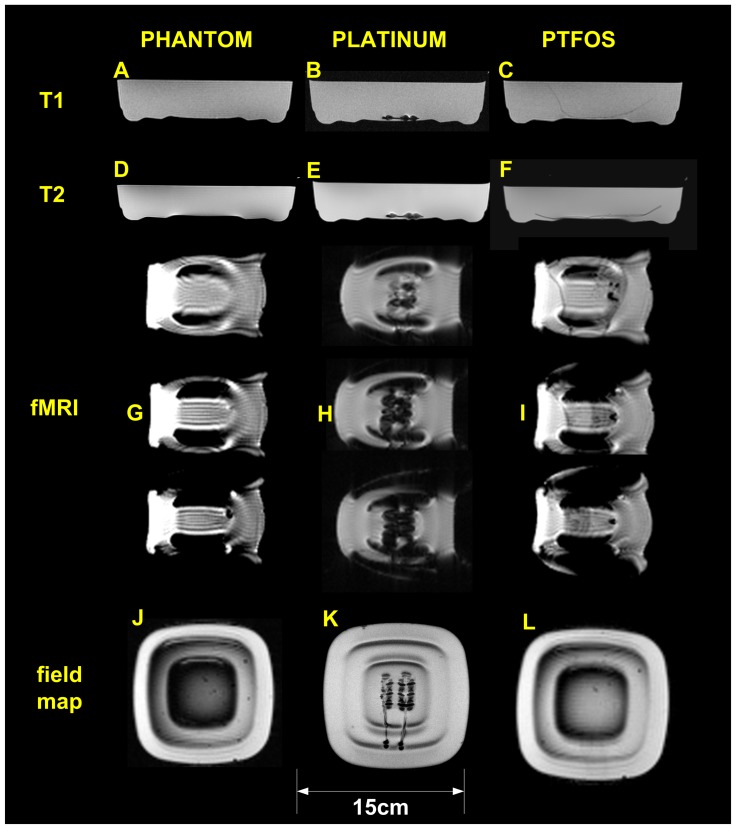
The Magnetic Resonance Imaging testing. T-1 weighted MPRAGE of the: (**A**) phantom only, (**B**) Platinum subdural grid and (**C**) PTFOS. T-2 weighted MPRAGE of: (**D**) phantom only, (**E**) Platinum subdural grid and (**F**) PTFOS. Three consecutive central slices of BOLD images of: (**G**) phantom only, (**H**) Platinum subdural grid and (**I**) PTFOS. Three consecutive central slices of BOLD images of: (**G**) phantom only, (**H**) Platinum subdural grid and (**I**) PTFOS. Fieldmap images of: (**J**) phantom only, (K) Platinum subdural grid and (**L**) PTFOS. Artifacts in the central column (platinum electrodes) are clearly visible in the central column.

MRI images were acquired in a 3Tesla (3T) Siemens Trio with a transmit/receive birdcage coil which is commonly used for imaging patients. The simple tissue phantom was doped with Gadolinium dissolved in a physiological solution with agarose and had dielectric and T1-weighted properties comparable to those of the human brain [12] consisting of a 0.7 L mixture of 0.9% Sodium Chloride (NaCl, Aqualite system, Hospira, Lake Forest, IL) saline solution containing 6.3 g of NaCl and 0.37 g of Gadobenate Dimeglumine (MultiHance, Bracco Diagnostics, Milano, Italy) in a disposable plastic square container (**Fig. 3**). The images collected were of the tissue phantom alone, the phantom with a standard electrode set, and the phantom with the PTFOS electrodes (**Fig. 3A–L**) to test for the presence/absence of MRI artifacts One hundred and twenty eight (128) T1 weighted slices 1mm thick were acquired using an MPRAGE sequence sagittally oriented, with 1×1×1 mm^3^ resolution, TR = 2.53 s/TE = 3.39 ms/TI = 1.1 s, FOV = 256×256 mm, dist factor = 50, flip-angle = 7°. Twenty-three T2 weighted slices 5.0 mm thick were acquired using a TSE sequence axially oriented with 0.8×0.8×5 mm^3^ resolution, TR = 5.21 s/TE = 81 ms, FOV = 200 mm, averages = 2, dist factor = 20, flip angle = 150°. In order to simulate a functional MRI sequence, twenty-four slices 5.0 mm thick were acquired using a BOLD sequence axially oriented with 3.1×3.1×5 mm^3^ resolution, TR = 2 s/TE = 30 ms, dist factor = 20, flip angle = 90°.

### Simulations

Standard metallic implants with high electrical conductivity inside the human body may lead to clear changes in the RF amplitude **B_1_** in the tissue under investigation, which may become more prominent at 3T [11] compared to lower fringe MRI fields. In order to study the changes in the **B_1_** field or the magnetic field at the Larmor frequency of approximately 127 MHz we performed electromagnetic Finite Differences Time Domain (FDTD) simulations with a geometry that modeled the phantom alone and the two different types of electrodes. These simulations closely matched the geometry (shape and dimensions) of the actual coil used in the MRI measurements (**Fig. 2A**), of the PTFOS electrode sets inside the phantom (**Fig. 2B**) and of the standard electrode set inside the phantom (**Fig. 2C**). The simulated coil was a birdcage coil with 16 spokes that was numerically tuned to match the physical coil matching the one used in the MRI measurements and the two feeds (i.e., with phases of 0° and 90°) were set to the voltage amplitudes reported by the scanner. For the PTFOS model, a rectangle of 100 mm×125 mm represented the Gelfilm substrate, on which the different types of PTF inks are laid. On top of this substrate a second rectangle 5 mm×35 mm represented the electrode lead that was a PTF either in silver (i.e., Ag) or in carbon (i.e., C). Finally the tip of the electrode was a rectangle 5 mm×10 mm with silver PTF electrical characteristics. The simulations also included a geometrical model (**Fig. 2C**) of the standard electrode set in which the electrodes were placed 10 mm apart and each electrode had a 5 mm diameter with a total of nine electrodes. The silicone substrate had dimensions of 18×80 mm.

The MRI coil was modeled as a quadrature-driven bandpass birdcage RF transmit/receive coil with an external shield of 400 mm in diameter, with 16 perfect electrically conductive (PEC) strip lines of 270 mm in length and 13.2 mm in width and disposed with circular symmetry (diameter 270 mm). The PEC striplines were connected at each extremity by two end-rings (diameter 270 mm) and 16 rungs located in the middle of each wire. Each end-ring of the birdcage coil was tuned with 16 capacitors with a value estimated by using the Circular Birdcage Builder (PennState Hershey College of Medicine, Hershey, PA) at 126.77 MHz with a value of 0.68pF. The bottom end-ring has two ports which were each matched with an inductor in series of 16.581 nH, a value estimated by using QuickSmith (http://www.nathaniyer.com/index.html, Santa Clara, CA). Each leg has a tuned capacitor of 1 μF in the middle (**Fig. 2A** **)**, a value also estimated by using the Circular Birdcage Builder. The RF coil model was finely tuned using s-parameters estimated by FDTD with a human model [13] at resonant frequency of 126.77 MHz. Each port of the coil was driven by two sinusoidal voltage feeds with amplitude given by the scanner adjustments (119.1 V for no electrodes, 119.3 V for platinum and 120.7 V for PTF), 126.77 MHz and a 90^o^ phase-shift between two ports and was loaded with a 50 Ω resistor in parallel to the generator.

**Figure 4:**
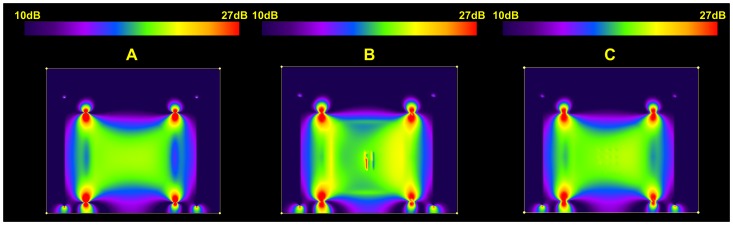
Results of the electromagnetic simulations. The **B_1_** field distribution in a plane inside the head coil and phantom containing: (**A**) no electrodes, (**B**) platinum and (**C**) PTFOS electrodes. The 0dB (red) scale corresponds to a reference value of 3.8 10^−7^ T.

Local **B_1_** fields (**Fig. 4A**–**C**) were computed with FDTD simulations using commercially available software (XFdtd 7.2.2, Remcom Inc., State College, PA). The overall volume used in the calculation was 107,616,600 mm^3^ with 3×3×3 mm^3^ isotropic Yee cells [14] to match the MRI measurements; however simulations at 1×1×1 mm^3^ isotropic have been performed as well to better capture the small geometric details of the commercial set with only minimal changes in the results. The simulation results are displayed in the xz plane of the electrodes.

**Figure 5:**
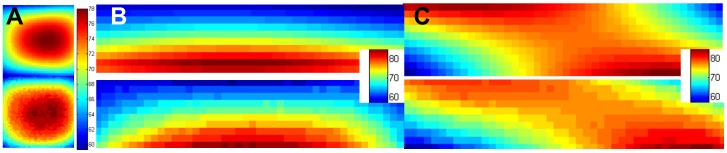
Validation analysis of the electromagnetic simulations. Numerical estimation (top) vs. AFI measurements (bottom) of the flip angle in the: (**A**) xy (**B**) xz and (**C**) yz planes.

Three numerical simulations were performed on the coil model: without electrodes, with PTFOS electrodes and with platinum electrodes. The dimension of the container was 210×210×39 mm^3^. Given the cell size was 1 mm^3^, the time step used to ensure FDTD stability from the Courant condition was 1.92 ps, for a total of approximately 50,000 time steps, corresponding to over 100 cycles covered and a steady-state reached after approximately 0.29 μs. Ten perfectly matching layers were used for boundary conditions [15]. All boundaries are set to absorbing condition. The PTFOS electrode sets used the following materials: 118–43 (silver, conductivity  = 400 S/m) and 119–28 (carbon, conductivity  = 0.05 S/m) measured with a network analyzer retrofitted with an impedance testing kit. The simulations were validated by comparing measured and numerically estimated values of flip angle (FA) inside the phantom (**Fig. 5**). The MRI measurements were performed using the actual FA imaging method [**16**] and the simulations provided the **B_1_** field that was used to estimate the flip angle that was then compared to the measured flip angle. Simulations were performed on a Precision T7500 (DELL, Round Rock, TX) with 48 GB of RAM and a graphic processor unit (Tesla C1060, NVIDIA, Santa Clara, CA) and lasted approximately 6 hours and 424 minutes. The electromagnetic field was generated by a birdcage antenna with currents supplied by two feeds (0° and 90°). Both feeds were driven by two 126.77 MHz sinusoidal generators with 1 mW power (V_f_ = 0.637 V and I_f_ = 12.4 mA) for the two models: the platinum and the PTF.

### Electrical Impedance Measurements

**Figure 6:**
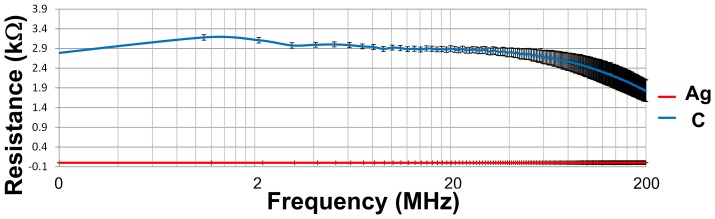
Electrical Impedance spectroscopy study of the traces. Resistivity of the PTF traces from 100 Hz to 200 MHz.

Ten measurements of conductivities from 200 kHz to 200 MHz were taken with a Network Analyzer (HP5061B NA with 100 kHz to 1.5 GHz band, Agilent technologies, Santa Clara CA) with a test fixture (HP16093A, Agilent technologies) in ten different trace positions and presented in terms of mean ± standard deviation (n = 3) (**Fig. 6**). The system was calibrated using both the standard 1-port calibration and the port extension compensation, using open/short/50 Ω loads and measurements were performed by setting the power to 9 dBm. The trace width was 2.54 mm, the sample length was 7 mm, typical thickness was 25 µm, and resistivity at 126.77 MHz was 2.26 kΩ for the C ink and 0.329 Ω for the Ag ink. The resistivity was 0.12 S/m for the 119–28 or the carbon based ink, 824 S/m for the 118–43 or the silver based ink and the Gelfilm (**Table 2**).

**Table 2:** Electrical properties and mass density of the electrodes.

Material	Conductivity (S/m)	Relative Permittivity	Density (kg/m^3^)
118–43	824	4.2	2,020
119–28	0.12	4.2	1,200
Gelfilm	0.33	10,000	8,700

### Microvolt Measurements and ECS-type stimulation

**Figure 7:**
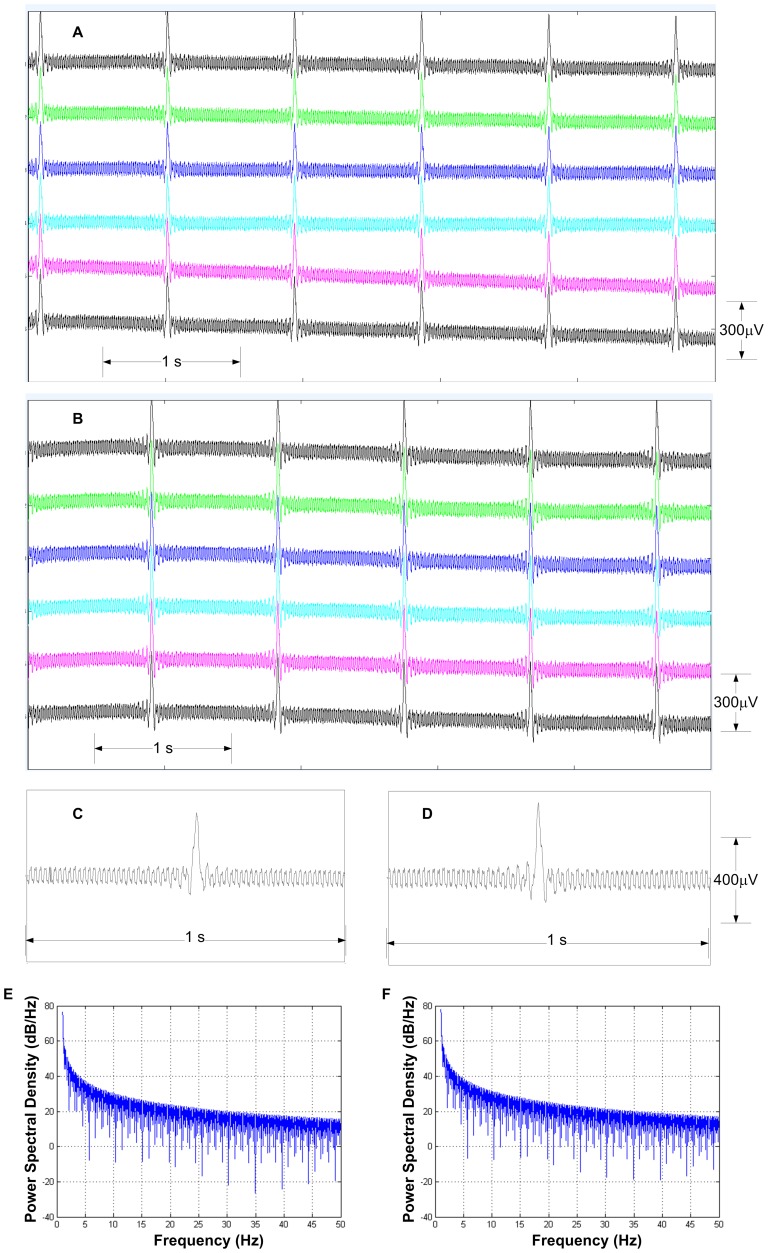
Recordings studies of the PTFOS electrodes set. Comparison between electrical recording of a Sin(x)/x function with amplitude of 300 µV of a stainless steel set (**A** and expanded in **C**) and the PTFOS (**B** and expanded in **D**). Comparison between power density spectrum (PSD) of silent (only noise) recording of a stainless steel set (**E**) and the PTFOS (**F**).

**Figure 8:**
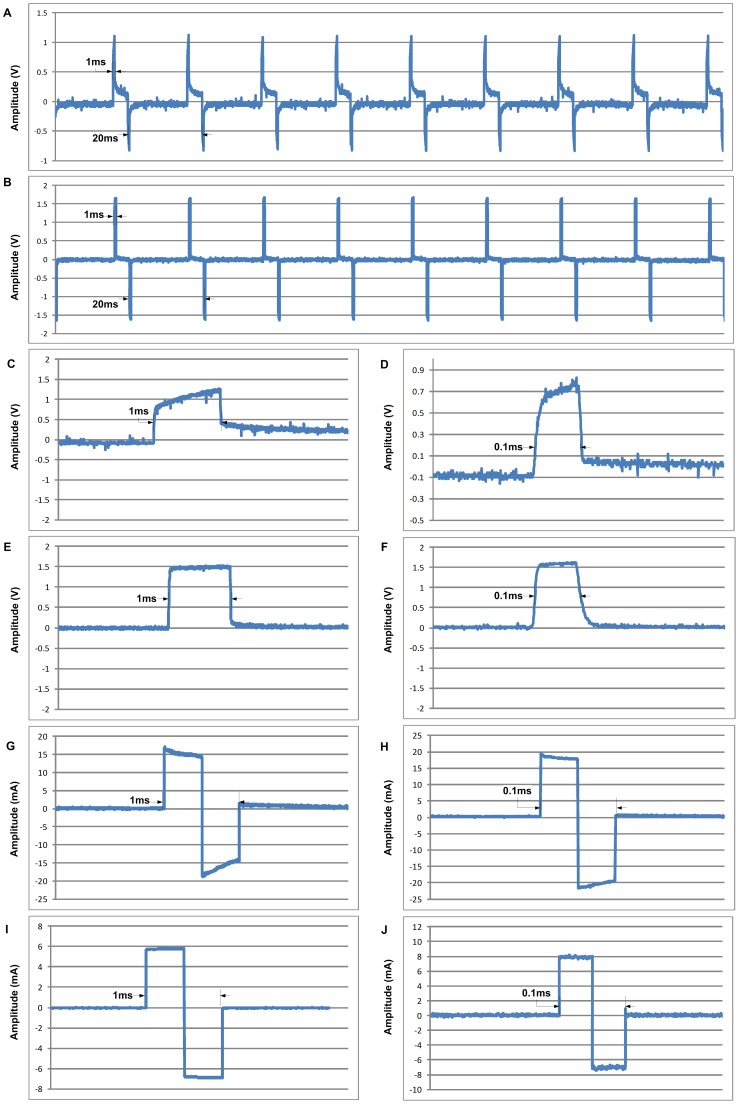
Stimulation studies of the PTFOS electrodes set. Comparison between 50 Hz current stimulation with a pulse duration of 1 ms for stainless steel set (**A** and expanded in **C**) and the PTFOS (**B** and expanded in **E**) and 0.1 ms for stainless steel set (**D**) and the PTFOS (**F**). Comparison between 50 Hz voltage stimulation with a pulse duration of 1 ms for stainless steel set (**G**) and the PTFOS (**I**) and 0.1 ms for stainless steel set (**H**) and the PTFOS (**J**).

Two types of measurements were performed on the MRI phantom described above to compare the PTFOS with a set of standard 16 electrode stainless steel subdural grid with respect to microvolt recording and ECS-type stimulation. In the microvolt recording experiment, a set of two gold electrodes (Grass Technologies, West Warwick, RI) were immersed in the phantom and were connected to a custom made adapter to convert to a Sub Multi Assembly (SMA) connector or a particular type of RF connector. The SMA connector was plugged in to a 60 dB attenuator (Mini-Circuits, Brooklyn, NY) and then was plugged into a signal generator (AFG3021B, Tektronix Inc., Beaverton, OR). The PTFOS set was bonded to nine microwires (AWG 34 (12–1234, Philmore, LKG Industries, Rockford, IL) using an Ag conductive epoxy (8331–14G, MG Chemicals, Surrey, B.C., Canada) and secured mechanically with a layer of light cured adhesive (3972, Henkel Loctite Corp., Rocky Hill, CT). The opposite end of each microwire was then soldered to a DB37 connector and a similar procedure was followed for the stainless standard subdural set. The 9-channel waves (**Fig. 7**) were recorded using a custom made adapter to connect the DB9 to our custom-made MRI compatible low noise EEG system High Field 1 (HF-1) [17], and the peak 300 μV amplitude and 1 Hz sync shape of the waveform were selected to simulate stimulation artifact of a ECS stimulation. In the noise recordings, the background noise was due to line (i.e., 60 Hz) noise and the instrument and resistive traces noises were not significant (<1 μVpp). The raw traces were saved by HF-1 and read into MATLAB (Mathworks Inc., Natick, MA) and plotted. In the ECS stimulation experiment (**Fig. 8**), two channels were selected from the PTFOS with a standard 16 electrode stainless steel subdural grid. The two channels were connected to the signal generator but one of the two channels was first connected to a 100 Ω resistor and the voltage on the resistor was recorded by a digital scope (DPO 3012, Tektronix). The 60 Hz stimulation bipolar pulse was selected to exceed the usual maximum current used in ECS (i.e., 10 mA we tested with the maximum allowed by the function generator of 30 mA and typical pulse width range of 0.1 to 1 ms). These pulses were generated by the signal generator for the voltage source case and the signal generator was reprogrammed using the proper software tool (arbexpress, Tektronix). Furthermore for the current source case, the pulses were generated by our EIS system [18], reprogrammed for these experiments using Labview (National Instruments Corp., Austin, TX). In the voltage source case, the current measurements were very noisy mostly because of line (i.e., 60 Hz) noise. In order to correct for the noise, the scope was programmed to average with the maximum number of averages allowed by the instrument (i.e., 256) time locked to the 50 Hz stimulus.

## Results

### MRI Measurements

The MRI properties of the PTFOS electrode set were compared to the standard set and to no electrodes in a phantom MRI study with clinical MRI sequences. The top row of **Fig. 3** shows T1-weighted images, the second row shows images of turbo spin echo T2-weighted images, the third row shows three fMRI slices (T2* epi), and the fourth row shows images of field map. The first two rows are taken with MRI sequences commonly used in clinical brain MRI and the third row is a typical echoplanar (epi) imaging fMRI sequence. These images represent the phantom alone (**Fig. 3**, left column), with the standard electrode set (**Fig. 3**, middle column), and with the PTFOS electrodes (**Fig. 3** right column). The artifacts generated by the standard set are clearly visible as a loss of signal with a circular shape generated by the wires in the top two central rows of the T1 and T2-weighted images (**Fig. 3B and 3E**). In the case of the PTFOS electrodes there is no artifact nor loss of signal but differences are observed in the contrast signal of the Gelfilm substrate compared to just the phantom alone. Similarly for the BOLD images on the third through fifth rows, signal loss is seen with the 9-channel platinum electrodes (**Fig. 3H**) and not with the PTFOS electrodes (**Fig. 3I**) whose appearance is similar to the phantom alone (**Fig.** **3G**). The artifacts from the platinum electrodes extend more broadly than the geometrical dimensions of the electrodes/wires, as solids have very short T2, too short to be visible in a conventional MRI. In the case of the PTFOS electrodes there is no artifact nor loss of signal but the different BOLD slices with respect to the phantom alone are to a different T2* contrast signal of the Gelfilm substrate compared to the phantom alone. Finally for the field map images in the last row, the signal loss was clearly present in the platinum electrodes (**Fig. 3H**) and not in the PTFOS electrodes (**Fig. 3I**) or the phantom alone (**Fig. 3G**). The proposed PTFOS electrodes carry a normal MRI signal since the Gelfilm substrate is a hydrated collagen or a biological tissue and the electrodes/traces are only a small fraction of a voxel (i.e., ∼5 µm). All these results confirm that the signal loss was present only for the phantom with the standard electrodes, since the proposed PTFOS electrodes contain only a fraction of metal (i.e., Ag/polymers) or no metal at all (i.e., carbon based traces) compared to the wires of the standard set (i.e., solid metal). These metals have magnetic susceptibility dissimilar to the surrounding tissue therefore causing the observed susceptibility artifact or signal loss. The two main sources of MRI artifact noise responsible for the observed signal loss are susceptibility artifacts and the **B_1_** field inhomogeneity. In order to understand the relative contribution of these two noise factors, we performed electromagnetic (EM) simulations. In these studies we investigated the amplitude of the **B_1_** field, a much less investigated source of artifacts, in the tissue surrounding the implant during a radio frequency (RF) pulse used for imaging in MRI [11].

### FDTD Simulations

In general, the RF pulse will induce currents in the tissue surrounding the metal implant and the current amplitudes will depend on the wire position, orientation, and length inside the coil. In turn, these currents induce a **B_1_** field as shown in the simulations (**Fig. 4A–C**). These currents may induce MRI artifacts that may lead to local **B_1_** amplification and increased regions of signal loss [19]–[22]. The simulations predicted a **B_1_** field of approximately 2 10^−9^ T for the PTFOS electrodes (**Fig. 4C**) compared to the peak of 3.610^−9^ T for the platinum or standard set (Fig. 4B), or a peak twice as high. Such large peaks in the **B_1_** field perturb the RF homogeneity near the metal electrode/wires by superposition, where the resulting **B_1_** field will be the superposition of the applied and the induced fields [23], [24]. These large peaks occur only in the **B_1_** field images with platinum traces enhancing the observed signal loss effect (**Fig. 4B**) whereas no **B_1_** field peaks were observed with the PTFOS electrodes (Fig. 4C) [16].

The FDTD validation results (**Fig. 5A–C**) show that the FA estimated with the FDTD simulations was within 10% of the FA measured with the actual flip angle imaging (AFI) method. Further numerical corrections to the AFI can improve even further the accuracy of the FA measurements with an even lower systematic error than the FDTD simulations [25].

The simulations relied on precise geometrical information of the coil, phantom, and electrodes. However, these simulations also require a very precise estimation of all the dielectric constants in the various models. When PTF are studied with EM simulations and since the binders used in PTF are polar, knowledge of the dielectric properties of the inks at the Larmor frequency of interest (∼127 MHz) is required. Binders serve to bind the nano-particles and provide adhesion to the substrate, ensuring the necessary viscosity (or flow) as a requirement for transfer of the ink from the press to the substrate, and contributing to the drying speed and resistance properties of an ink. The problem with polar binders is that these compounds have dispersive dielectric properties, with an electrical conductivity increasing at higher frequencies.

Since the PTF conductivity is most commonly specified by the manufacturer at only one frequency (i.e., low or DC) whereas the PTFOS electrodes are designed to function at the much higher MRI Larmor frequencies (64 MHz, 128 MHz and 300 MHz), we measured the resistivity to estimate the value to use in our FDTD simulations. The data are presented **Fig. 6**, and shows that the resistivity of both the carbon traces and silver electrodes are quite flat from 100 Hz to 200 MHz, even though there is a total 53% drop from the DC value. In all our simulations we adjusted the value of the dielectric properties of the PTFOS according to the electrical impedance spectroscopy measurements.

The PTFOS electrodes were tested for their ability to record μV signals as in the case of electrocorticography and were compared with the standard stainless steel electrodes. The results (**Fig. 7**) show that the PTFOS (**Fig. 7B and 7D**) are capable of recording μV signals with accuracy and stability of the traces similar to the standard electrodes (**Fig. 7A and 7C**).

The quality of the recordings from the two electrodes sets was compared using a stimulation function (i.e., Sinc function) that in shape and amplitude modeled the ECS response from the human cortex, typically a train of spikes [26]. The signals from the standard electrodes (**Fig. 7A and 7C**) and from the PTFOS (**Fig. 7** **B** and **7D**) appear to be identical. In particular, with respect to the shape, we estimated an average peak of the cross correlation function between the two of electrodes was 0.999994989±0.00000249. The cross-correlation function is a function similarity between one waveform and a time-shifted version of the other, as a function of this time shift and the maximum occurs when the two functions maximally overlap. The normalized cross-correlation function has a scale that runs from zero to one, with one indicating a shift that produces two identical functions and zero indicating a complete non-correlation. The signal to noise ratio (SNR) or the measure of signal strength relative to background noise was also computed. The ratio is usually measured in decibels (dB) and we measured SNR inside a band of 0–40 Hz to avoid the 60 Hz line noise. The background noise consisted of noise from the instrumentation but also from the electrodes and from the physiological solution bath. The results showed that the two electrodes sets had a very similar SNR, in particular the standard electrodes had a SNR of 32.54dB and the PTFOS had slightly better SNR of 34.71 dB and was well above the SNR level (i.e., 13 dB) needed for subdural recordings [27].

In order further test if the PTF traces had unwanted noise characteristics [28], the noise characteristics of the electrodes was measured using the phantom by means of a statistical signal processing tool called the power spectral density (PSD) [29]. The PSD represents the power or energy function as function of frequency and has the dimensions of power or energy ratio in logarithmic units (dB) per hertz (Hz) and delineates how the power of the signal from the electrodes is distributed with frequency. In our experiments the power was defined as the squared value of the signal, as the actual power dissipated in the phantom solution just if that same signal was a voltage applied across the physiological solution. The PSD can help us understand the similarity or lack of similarity between two electrodes sets much better that showing the raw signal, which can never be truly similar as it is a random signal. The system in consideration is a linear system, so PSD is the cascade or product [30] of the following contributions: electrodes, physiological solution and instrumentation. Since the last two essentially remain the same across experiments, the differences in PSD really represent differences between the two electrode sets, unless the shape remains the same but it is scaled by different electrode geometry. The results show that the PSD of the standard electrodes was very similar to the PTFOS set (**Fig. 7E** vs. **7F**), where the PSDs were averaged over all times and channels with frequencies up to 50 Hz (i.e., the typical EEG bandwidth). The standard electrodes had a DC peak of 4.36±0.04 10^7^dB/Hz and the PTFOS had a 5.98±0.3 10^7^ dB/Hz and the 1 Hz peak was 2.29±1.2 10^4^ dB/Hz for the commercial set and the PTFOS had a 3.14 ±0.4 10^4^ dB/Hz peak. The slightly larger values were due to geometrical differences of the two set of electrodes as the distance, size and position were dissimilar. Thus we conclude that the two electrodes sets did not show any significant differences in terms of signal recording quality.

Finally, the PTFOS were tested for their ability to deliver currents with shapes and intensities similar those used in ECS when either current driven (**Fig. 8A–F**) or voltage driven (**Fig. 8G–J**). The PTFOS electrode set (**Fig. 8B, 8D and 8F**) was capable of delivering currents with similar shapes and amplitudes to the commercial electrodes (**Fig. 8A, 8C and 8E**) used in surgery at the two extreme pulse widths used in ECS (1–0.1 ms). Similarly, the PTFOS (**Fig. 8H and 8J**) similar shapes and amplitudes to the commercial electrodes (**Fig. 8G and 8I**) when driven by a voltage source. In both sets of experiments, the PTFOS response was a signal with a more ideal rectangular shape, resembling the original square pulse of the current or voltage sources.

The electrical and physical characteristics of the PTFOS traces did not change significantly after bathing the electrodes in a physiological solution for three months to simulate chronic implantation conditions, while the Gelfilm substrate was gradually broken down resulting in complete detachment from the traces while leaving the traces, themselves, intact even after 12 months of bathing.

## Discussion

The development of novel implantable electrode technology affords important innovations in functional neurosurgery and electrophysiology [31]. Traditional subdural electrode sets still have conductivities larger than 10^5^ Ω^−1^m^−1^ necessary to conduct electrical currents for electrocortical stimulation or for EEG recordings for epileptic foci localization. Such large conductivities may provoke generation of RF-induced eddy currents when patients with grids undergo MRI, potentially generating local heating in the electrodes that may injure the surrounding tissue [32], [33]. The large artifact that we observed in the T1 weighted image of the conventional subdural grids has also been shown in epileptic patients who underwent MRI after intracranial implantation of grids [34]. Many neurosurgeons and neuroradiologists are hesitant to perform MRI examinations with implanted subdural grids for fear of electrode displacement, current induction, heating, or image artifact in the strong magnetic field. The PTFOS electrode set we have developed potentially addresses all the above concerns as shown by the results reported here and in safety studies of PTF materials we have used in scalp EEG/fMRI recordings [5], although safety studies specific to any proposed final implant design will need to be performed.

The flexible and thin nature of the electrodes should make them well suited for chronic implantation. Because the brain surface is convoluted, achieving durable optimal contact across many electrodes can sometimes be difficult. Assessing whether this new technology performs as expected *in vivo* will require animal and later human studies. Even though the PTFOS are stretchable and flexible, the various PTF layers do not flake off because of a strong chemical bound between the binder of the various inks and the organic substrate. The PTF inks were deposited using pad printing, however other deposition techniques are available including: silk screen, inkjet, pen and photolithography [35]. The tight bounding was also achieved through the process of curing or hardening of the PTF coatings through polymerization or solvent evaporation. Given the stringent temperature limits on the Gelfilm, we selected a thermoplastic binder that would allow curing at very low temperatures and the PTFOS were ready to use after oven curing at 50°C for 3 hours.

The PTFOS electrodes could also be used for electrocortical or other neural stimulation in a more permanent or chronic setting, although additional studies are needed to examine the effect of long term continuous electrical stimulation in chronic implantation. Similar types of conductive polymers have been employed for neural stimulation [36], and Ag/AgCl epoxy-based electrodes have been studied in neural tissue stimulation [37]. This other work supports our results indicating that the PTFOS perform as well as the standard electrode set. In chronic implantations, it may be advantageous to employ less neurotoxic nano-particles than silver such as gold, however Ag/AgCl PTF electrodes not in contact with neurons were found to be non-toxic [38]. The major requirements for the dielectric layer needs are of low permeability to water to prevent electrical current leaks and biocompatibility. Biocompatibility studies addressing the impact of this type of electrode using different conductors and implanted in different locations of the body will need to be carried out. A similar flexible and absorbable electrode set has been reported [39], but with an important difference compared to the PTFOS. The other electrodes consist of traces/electrodes made of thick metal (15 μm of Cr/Au) therefore with very high conductivities non-suitable for MRI as discussed above.

Finally, there are a number of risks associated with conventional electrodes which may be mitigated by our approach. Concerns about risks such as cortical heating have generally precluded obtaining MRI in patients with implanted electrodes. We have successfully employed PTF technology similar to the PTFOS introduced in this work to reduce the risks of heating for scalp EEG and the resulting EEG cap (Inkcap) was truly RF transparent [5]. The InkCap was tested with temperature measurements on an electrically conductive phantom with a realistic human head shape while running functional MRI (fMRI) and high power structural recordings at 7T. The results showed [5] that the temperature increase at an electrode (C_z_) was over 6°C for standard EEG electrode set more than the Food and Drug Administration (FDA) limit of 1°C. While using the InkCap, the value of temperature increase at any electrode was within the FDA limit of 1°C. Since one of the inherent risks in placing a chronically implanted electrophysiological system is the inability to obtain future MRIs with consequent limitation of diagnostic or therapeutic options, the approach presented here may improve the overall risk benefit ratio such that many more patients could have implanted electrophysiological devices. Although the present data does not support any decrease in the risks of intracranial application, it is possible that the thinner profile and flexibility of this system would decrease risks of mass effect, inflammation (aseptic meningitis), and hemorrhage.

In summary we have introduced and a new type of MRI compatible intracranial electrode (PTFOS) based on carbon nano-particle polymer thick film technology. In contrast to the large artifacts exhibited with standard electrodes sets, these PTFOS electrodes exhibited no artifact due to the reduced amount of metal and conductivity of the electrode/trace ink. The enhanced MRI image quality did not compromise the electrical performance of the electrodes with respect to both stimulation and recordings compared to a standard commercial set.
